# Complete genome of *Acinetobacter calcoaceticus* AC001_UM from Red River soil banks in Winnipeg, Manitoba, Canada

**DOI:** 10.1128/mra.01122-23

**Published:** 2024-04-18

**Authors:** Ruwani L. Wimalasekara, Ellen M. E. Sykes, Dawn White, Chris Rathgeber, Ayush Kumar

**Affiliations:** 1 Department of Microbiology, University of Manitoba, Winnipeg, Manitoba, Canada; California State University San Marcos, San Marcos, California, USA

**Keywords:** ACB complex, *bla*_*OXA-268*_

## Abstract

We report the whole-genome sequence and antibiotic-resistance gene profile of an *Acinetobacter calcoaceticus* isolate, designated AC001_UM, taken from soil along the Red River in Winnipeg, Manitoba, Canada. The genome comprised 3,916,544 nucleotides (GC content: 38.7%). Antibiotic-resistance gene analysis revealed a class D β-lactamase and three efflux pump families.

## ANNOUNCEMENT

*Acinetobacter calcoaceticus*, part of the *Acinetobacter calcoaceticus-Acinetobacter baumannii* complex, is ubiquitous in nature but is also emerging as a major healthcare challenge due to antibiotic resistance (1). We isolated an *A. calcoaceticus* that we designated AC001_UM from the banks of the Red River in Winnipeg, Canada, and sequenced its genome to gain insight into its resistome.

**Fig 1:**
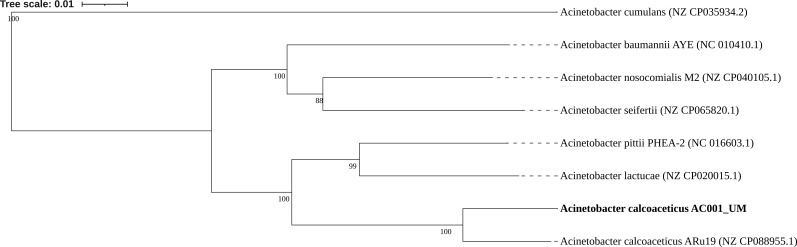
Phylogenetic dendrogram of *A. calcoaceticus* AC001_UM and *Acinetobacter* spp. The tree was inferred with FastME 2.1.6.1 (12) based on genome sequences analyzed using the Genome BLAST Distance Phylogeny (GBDP) method. Branch lengths are scaled in terms of GBDP distance formula d5, and numbers above the branches indicate GBDP pseudo bootstrap support values of >60% from 100 replications, with an average branch support of 97.8%. The tree was rooted at the midpoint.

Isolation involved plating 0.1 mL of suspension (1 g soil/1 mL saline) on MacConkey agar (Oxoid, UK) and incubating at 37°C for 24 hours. Distinct colonies were re-streaked on Tryptic Soy Agar (Oxoid) and tentatively identified as *Acinetobacter* spp. using the API 20E system following the manufacturer’s instructions (Biomerieux, France). DNA was extracted with an EZ-10 Spin Column Bacterial Genomic DNA Miniprep Kit (Bio Basic Inc, Canada). Unsheared and non-size selected DNA was used to generate libraries with SQK-LSK114 (Oxford Nanopore Technologies) and sequenced with the MinION FLO-MIN112 (Oxford Nanopore Technologies, UK). Default parameters were used for all sequence processing unless otherwise noted. Base-calling and filtering low-quality reads (cut off-Q9) were achieved using Guppy v.6.3.7 (Oxford Nanopore Technologies). Sequence adapters were trimmed with Porechop v.0.2.1 (2). A total of 483,964 reads (length N_50_ of 9685) were assembled *de-novo* with Flye v.2.9.1 (3) and verified with Bandage v.0.9.0 (4). Sequencing quality was evaluated with QUAST v.5.0.2 (5); the genome consisted of a single contig (length 3,916,544) with a GC content of 38.7%, an N_50_ of 3,916,544 bp, and 700× coverage. Genome completeness was verified with CheckM v.1.0.11 (6) (99.4% completeness, 1.14% contamination) and whole-genome alignment with reference genome *A. calcoaceticus* Aru19 (NZ_CP088955.1) using progressiveMauve v.2.4.0 (7). Annotation with NCBI Prokaryotic Genome Annotation Pipeline v.6.5 (8) revealed 3,646 protein-coding genes, 95 RNA genes (18 rRNAs, 73 tRNAs, and 4 ncRNAs), and 370 pseudogenes. Our isolate was confirmed as *A. calcoaceticus* with Fastani v.1.33 (9) and shared 96.2% nucleotide identity with the reference strain Aru19. The phylogeny of AC001_UM was verified using whole-genome taxonomy-based analysis in Type Strain Genome Server (10) and visualized using iToL (11) (Fig. 1).

Resistance genes were identified with the Comprehensive Antibiotic Resistance Database (13) using RGI *main* with strict and perfect parameters (Table 1). Of note was the class D β-lactamase *bla_OXA-268_* implicated in carbapenem resistance (14); BLASTn against the NCBI RefSeq genome database (https://www.ncbi.nlm.nih.gov/datasets/taxonomy/471/) revealed that *bla_OXA-268_* is prevalent in 93% *A*. *calcoaceticus* (15). A component of the AdeFGH multidrug efflux pump, *adeG,* was also identified (16). Using RefSeq, we found a single nucleotide polymorphism in AC001_UM at position 356 (356delC) of *adeH* resulting in a frameshift mutation and predicted truncated protein. It will be intriguing to explore how this mutation might influence the antibiotic susceptibility profile of AC001_UM; however, as this assembly was generated using Nanopore-only data it is possible that this frameshift is artifactual. In conclusion, the *A. calcoaceticus* AC001_UM genome offers valuable genetic insights for future studies on antibiotic resistance in *Acinetobacter* spp.

**TABLE 1:** Antibiotic resistance genes of *Acinetobacter calcoaceticus* AC001_UM identified from CARD analysis

Antibiotic resistance gene	Locus tag	Antibiotic resistance gene family	CARD accession	Percent sequence identity
*adeJ*	Q4S33_00240	Resistance-nodulation-division transporter	ARO:3000781	96
*bla_OXA-268_*	Q4S33_12415	OXA β-lactamase	ARO:3001724	100
*amvA*	Q4S33_15630	Major facilitator superfamily transporter	ARO:3004577	96
*abeS*	Q4S33_17045	Small multidrug resistance family transporter	ARO:3000768	94
*adeG*	Q4S33_17060	Resistance-nodulation-division transporter	ARO:3000777	98

## Data Availability

The BioProject, BioSample, and Sequence Read Archive numbers for AC001_UM are PRJNA996987, SAMN36661286, and SRR26398677, respectively. The GenBank accession of the genome is CP130683.1.
